# The FOXG1/FOXO/SMAD network balances proliferation and differentiation of cortical progenitors and activates *Kcnh3* expression in mature neurons

**DOI:** 10.18632/oncotarget.9545

**Published:** 2016-05-21

**Authors:** Riccardo Vezzali, Stefan Christopher Weise, Nicole Hellbach, Venissa Machado, Stefanie Heidrich, Tanja Vogel

**Affiliations:** ^1^ Department of Molecular Embryology, Institute of Anatomy and Cell Biology, Faculty of Medicine, University of Freiburg, Freiburg, Germany; ^2^ Faculty of Biology, University of Freiburg, Freiburg, Germany

**Keywords:** TGFβ, neurogenesis, transcriptional control, cerebral cortex, atypical Rett syndrome, Gerotarget

## Abstract

Transforming growth factor β (TGFβ)-mediated anti-proliferative and differentiating effects promote neuronal differentiation during embryonic central nervous system development. TGFβ downstream signals, composed of activated SMAD2/3, SMAD4 and a FOXO family member, promote the expression of cyclin-dependent kinase inhibitor *Cdkn1a*. In early CNS development, IGF1/PI3K signaling and the transcription factor FOXG1 inhibit FOXO- and TGFβ-mediated *Cdkn1a* transcription. FOXG1 prevents cell cycle exit by binding to the SMAD/FOXO-protein complex. In this study we provide further details on the FOXG1/FOXO/SMAD transcription factor network. We identified ligands of the TGFβ- and IGF-family, *Foxo1*, *Foxo3* and *Kcnh3* as novel FOXG1-target genes during telencephalic development and showed that FOXG1 interferes with *Foxo1* and *Tgfβ* transcription. Our data specify that FOXO1 activates *Cdkn1a* transcription. This process is under control of the IGF1-pathway, as *Cdkn1a* transcription increases when IGF1-signaling is pharmacologically inhibited. However, overexpression of CDKN1A and knockdown of *Foxo1* and *Foxo3* is not sufficient for neuronal differentiation, which is probably instructed by TGFβ-signaling. In mature neurons, FOXG1 activates transcription of the seizure-related *Kcnh3*, which might be a FOXG1-target gene involved in the FOXG1 syndrome pathology.

## INTRODUCTION

During neuronal differentiation progenitor cells are instructed according to a precise spatial and temporal plan, and specific signals control the equilibrium between proliferation and differentiation. Among these signals are extrinsic cues such as growth factors or activators of intracellular signaling pathways. Since proliferation and differentiation take place simultaneously in developing organ systems, cellular interpretation of such signals has to occur cell autonomously, for example through activity of specific transcription factors. Transforming growth factor β (TGFβ) is an extrinsic cue implicated in neuronal differentiation of cortical progenitor cells (CPCs) [1, 2]. It has 3 different isoforms (TGFB1, 2, and 3) and is a member of the TGFβ superfamily, including Bone morphogenetic proteins (BMPs) and Activins. Canonical TGFβ-signaling occurs *via* SMAD-dependent pathways, in which the receptor complex phosphorylates R-SMAD (SMA- and MAD-related protein) 2 and/or 3. Phosphorylated SMAD2 and 3 translocate to the nucleus together with SMAD4 [3]. SMAD proteins bind to diverse cofactors. SMAD/cofactor complexes activate or inhibit context-dependent transcription of a variety of target genes, which is apparent through the diversity of processes controlled by TGFβ [4–6].

During embryonic neurogenesis, TGFβ exerts antiproliferative and differentiating effects on neuronal progenitor cells [1, 7, 8]. TGFβ-signals lead to cell cycle arrest in G1 phase by transcriptional activation of the cyclin-dependent kinase inhibitors *Cdkn1a* and *Cdkn2b,* as well as repression of the myelocytomatosis oncogene (*cMyc)* and inhibitor of DNA binding 1, 2 and 3 (*Id1-Id3)* [9–11]. Forkhead box O (FOXO) proteins are cofactors of SMAD3 and SMAD4 in the TGFβ-induced formation of a *Cdkn1a*-activation complex [7]. FOXO proteins are important in the control of cell and organismal growth, development, metabolism and longevity. The phosphatidyl inositol 3-kinase (PI3K) growth-promoting pathway negatively controls FOXO factors through thymoma viral proto-oncogene (AKT)-mediated phosphorylation of FOXO proteins, which prevents their translocation to the nucleus [12].

Another FOX family member, FOXG1, opposes the activity of SMAD/FOXO-complexes by preventing transcriptional activation of *Cdkn1a* through a non-competitive, direct binding of FOXO3 in the FOXO/SMAD complex [7, 13]. Absence of FOXG1 during mouse embryonic development leads to death at birth due to hypoplasia of cerebral hemispheres [14]. In CPCs it promotes self-renewal of neural precursors and antagonizes neuronal differentiation [14–17]. FOXG1 expression is dynamic during cortical development whereby it is transiently downregulated when progenitors enter neuronal differentiation. The re-expression of FOXG1 in differentiating neurons is necessary for correct integration into the cortical plate [18]. The interference of FOXG1 with TGFβ- and FOXO-mediated cell cycle exit might be responsible for its inhibition of neuronal differentiation. However, as the biochemical data that described the role of the FOXG1/FOXO/SMAD transcriptional complex in *Cdkn1a* expression comes from keratinocytes [7], this notion has still to be proven in CPCs. Regulation of *Cdkn1a* expression by TGFβ, FOXO3 and FOXG1 might also be important for the differentiation of Cajal-Retzius (CR) neurons [19]. CR cells are among the earliest born neurons in the developing cerebral cortex [20–22] and are generated from different telencephalic regions, some of which do not express FOXG1 [23, 24].

Despite a substantial body of data, the functional role of the FOXG1/FOXO/SMAD transcription factor network in the cerebral cortex is mostly correlative [8, 19, 25] and several open questions remain. Firstly, FOXG1 and FOXO proteins might be a node of intersection between TGFβ- and IGF-signaling pathways. In contrast to this view, we recently reported that IGF1-signaling activates cell proliferation in early cortical development (E13.5), whereas TGFβ-signaling is mainly active at later stages (E16.5) [2]. Hence, FOXG1 and FOXO proteins might be cofactors that are implicated in different developmental responses to IGF1- and TGFβ-signals rather than nodes of intersection. Secondly, it is unclear whether expression of *Cdkn1a* or FOXO proteins is sufficient to stimulate neuronal differentiation. Thirdly, further target genes apart from *Cdkn1a* in CPCs or in mature neurons might be controlled by FOXG1/FOXO/SMAD transcription factors. Hence, we studied the FOXG1/FOXO/SMAD network in CPCs of different developmental stages and in different mouse models. Our analyses revealed that (1) FOXG1 impaired TGFβ-induced neuronal differentiation in early developmental stages, i.e. E13.5; (2) FOXG1 blocks transcription of *Cdkn1a, Tgfβ, Foxo1* and *Foxo3*; (3) expression of *Cdkn1a* is activated by FOXO1; (4) neither CDKN1A, FOXO1 or FOXO3 can stimulate neuronal differentiation autonomously; and (5) *Kcnh3* is a novel neuronal FOXG1-regulated target gene which might be of clinical relevance in atypical Rett syndrome.

## RESULTS

### FOXG1 antagonizes TGFβ-mediated neuronal differentiation at early developmental stages

**Figure 1:**
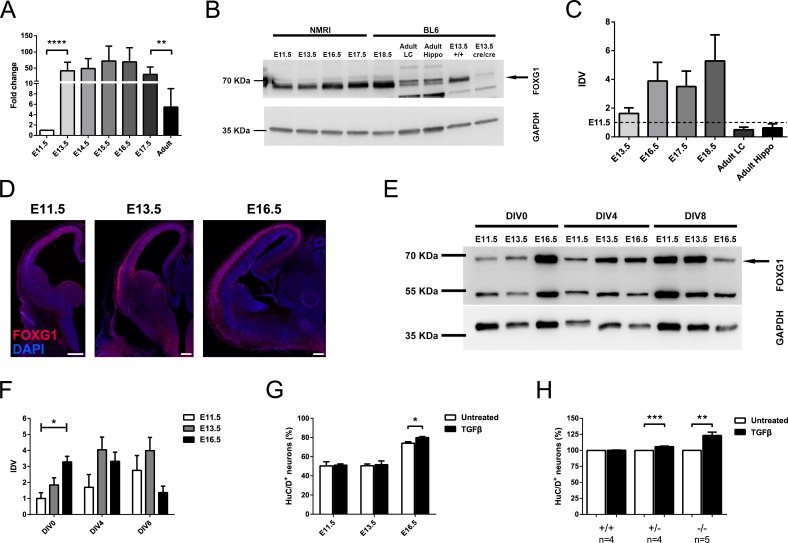
FOXG1 prevents TGFβ-mediated neuronal differentiation at early developmental stages **A.** qRTPCR analysis of *Foxg1* expression from E11.5 until adulthood revealed that *Foxg1* transcript levels increased significantly after E11.5 and remained stationary until they dropped at the adult stage. Values were expressed as fold change relative to E11.5 (indicated as 1). ****p < 0.0001; **p < 0.01; One-way ANOVA - Šidák's post-test comparing consecutive developmental stages; *n* = 3. **B.**, **C.** Immunoblot evaluation of FOXG1 expression in murine telencephalon at different developmental time points (B) and relative densitometric analysis (C) showed a mild but not significant increase in FOXG1 levels between E11.5 (dashed line) and E13.5. The peak of expression occurred between E13.5 and E17.5 and was decreased after E17.5 in lateral cortex (LC) and in hippocampus (hippo). Brain extracts from Foxg1^−/−^ animals were used as a negative control to correctly identify FOXG1 band (arrow). Statistical analysis: One-way ANOVA - Šidák's post-test comparing consecutive developmental stages; *n* = 3. **D.** Immunohistochemical staining showing temporo-spatial dynamics in expression of FOXG1 in murine forebrain at different developmental stages. Scale bar: 200 μm; *n* = 3. **E.**, **F.** *In vitro* assessment of FOXG1 protein levels on cortical cells obtained at E11.5, E13.5 and E16.5, and cultured until either DIV0, DIV4 or DIV8 (E), and their relative densitometric analysis (F). FOXG1 levels significantly increased between E11.5 and E16.5 in DIV0 cortical cells. CPCs from E11.5 and E13.5 show increased or unchanged FOXG1 expression, respectively, after 4 or 8 days in culture. FOXG1 in E16.5 cells drops at DIV8. *p < 0.05; One-Way ANOVA with Tukey's post-test comparing all pairs of columns within the same DIV group; *n* = 3. **G.** Evaluation of TGFβ-induced neuronal differentiation at E11.5, E13.5 and E16.5 through HuC/D immunocytochemistry. TGFβ treatment led to an increase in the percentage of HuC/D^+^ cells as compared to untreated control only at E16.5, while no significant effects were visible at E11.5 and E13.5. *p < 0.05; Student's *t*-test; *n* = 4. **H.** Evaluation of neuronal differentiation in E13.5 wild-type, *Foxg1*^+/−^ and *Foxg1*^−/−^ CPCs upon TGFβ treatment. HuC/D immunocytochemistry showed that partial or total loss of *Foxg1* renders cells responsive to TGFβ-induced differentiation. ***p < 0.001, **p < 0.01; Student's *t*-test. Immunoblot results (B-E) were normalized to respective GAPDH and shown as a ratio to the E11.5 stage (set as 1). All data are shown as mean±SEM. IDV: integrated density value.

*In vitro* cultivated CPCs from E16.5 mouse cerebral cortex differentiate upon a TGFβ stimulus, but this instructive effect is not observed in E13.5-derived cells [1, 2]. FOXG1 has the ability to prevent premature differentiation [16] and it antagonizes the TGFβ-pathway by inhibiting *Cdkn1a* transcription through association with the FOXO/SMAD4 complex, at least in keratinocytes [7]. Based on these observations, we hypothesized that altered expression levels of FOXG1 could be causative for the differences in responsiveness to TGFβ of E13.5 and E16.5-derived CPCs. Although FOXG1 has been studied to some extent, FOXG1 mRNA and protein expression during development has not yet been reported. We assessed FOXG1 expression in the telencephalon *in vivo* using reverse transcription-quantitative real-time PCR (qRTPCR) (Figure 1A), immunoblotting (Figure 1B, 1C) and immunohistochemistry (Figure 1D) at different developmental stages. On the transcriptional level, *Foxg1* expression increased significantly after E11.5 and remained on similar levels until the adult stage, where it declined significantly compared to embryonic stage E17.5 (Figure 1A). Expression changes on the protein level were slightly shifted to the later stage and the highest amounts of FOXG1 were identified in E16.5, E17.5 and E18.5 (Figure 1B, 1C). In the adult, less protein was detected in the lateral cortex (LC) and hippocampus (Hippo) compared to embryonic telencephalon of different stages. Immunohistochemistry of FOXG1 in forebrain sections of E11.5, E13.5 and E16.5 indicated that FOXG1 was expressed predominantly in progenitor cells at E11.5, whereas E13.5 forebrains expressed FOXG1 in progenitor cells and mature neurons. At E16.5 FOXG1 expression is confined mainly to mature neurons (Figure 1D). Together, these data indicated that the expression of FOXG1 is dynamic during development. FOXG1 expression thereby increased from E11.5 onwards, and coincided with increasing numbers of postmitotic neurons in the cortical plate. Hence, FOXG1 could indeed antagonize TGFβ-mediated neurogenesis during early development at E11.5 and E13.5. On the other hand, reduced levels of FOXG1 in E16.5 progenitor cells might render progenitors responsive to differentiative TGFβ-signals. Our recent reports on TGFβ-mediated neuronal differentiation are based on a culture paradigm where E13.5- and E16.5-derived cortical cells were cultured until day *in vitro* (DIV) 8 [2]. We therefore hypothesized that FOXG1 expression in CPCs might decline between E13.5 and E16.5 *in vitro*. We thus assessed FOXG1 expression in cultured CPCs that derived from different developmental time points (E11.5, E13.5 and E16.5) and were cultured for either 0, 4 or 8 DIV. Using immunoblotting of protein extracts we showed that FOXG1 expression levels of freshly dissociated cells increased during development as observed *in vivo* (Figure 1E, 1F). However, while cultivated cells from E11.5 and E13.5 increased or at least retained FOXG1 protein levels during the 4 and 8 day culture period, respectively, E16.5-derived cells expressed decreasing levels of FOXG1 after 8 days in culture (Figure 1E, 1F). To further address whether declining FOXG1 levels promote TGFβ-mediated neuronal differentiation, CPCs from E11.5, E13.5 and E16.5 were cultured for 8 DIV in the presence of TGFB1 from DIV2 onwards. Immunostaining and quantification of the neuronal marker HuC/D revealed that TGFβ treatment significantly increased the amount of HuC/D-positive cells at E16.5, but not at E13.5 or at E11.5 (Figure 1G). Finally, we treated E13.5-derived CPCs from *Foxg1-*deficient (Foxg1^−/−^), heterozygote (Foxg1^+/−^) and wild-type mice with TGFB1 and quantified cells positive for the neuronal marker HuC/D. Strikingly, TGFB1 treatment increased the numbers of HuC/D-positive neurons even in CPCs of E13.5, if the expression of FOXG1 is reduced, but not in wild-type CPCs expressing normal FOXG1 levels (Figure 1H).

We concluded that expression of FOXG1 impaired TGFβ-induced neuronal differentiation in a dose dependent manner at early developmental stages (E11.5 and E13.5).

### FOXG1 suppresses transcription of *Tgfβ*- and *Igf*-ligands, *Foxo1* and *Foxo3* transcription factors and *Cdkn1a in vivo*

**Figure 2:**
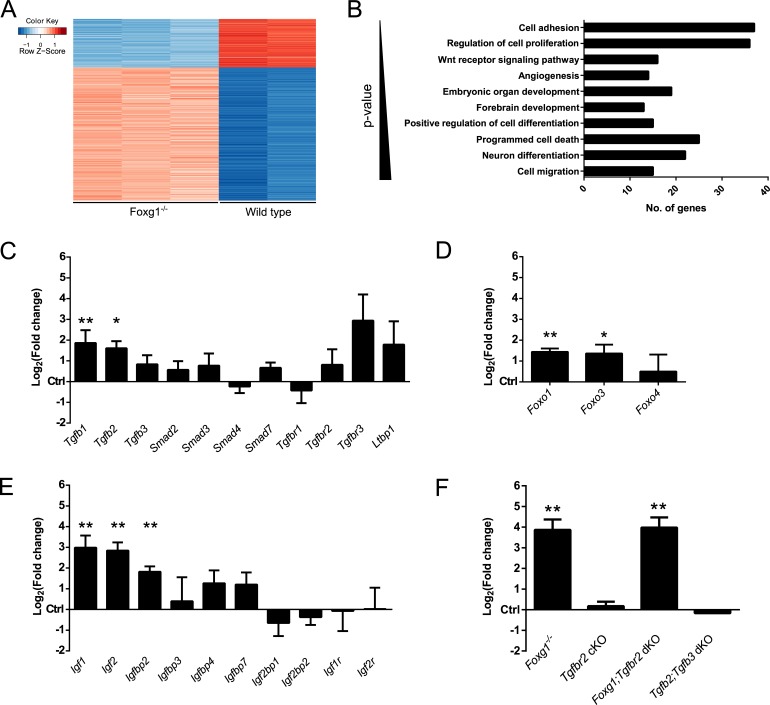
FOXG1 inhibits expression of *Tgfβ*- and *Igf*-ligands, *Foxo1*, *Foxo3* and *Cdkn1a* **A.** Transcriptome profiling of TGFβ-treated *Foxg1*^−/−^ (*n* = 3) and wild-type (*n* = 2) CPCs identified 586 differentially regulated genes. **B.** Bar chart showing the main biological processes in which microarray-identified genes are involved. This analysis was performed using DAVID. **C.**-**E.** Candidate genes identified through microarray analysis were validated by qRTPCR. Transcriptional expression of *Tgfb1* and *Tgfb2* (C), transcription factors *Foxo1* and *Foxo3* (D) as well as *Igf1* and *Igf2* (E) was significantly increased in E13.5 Foxg1^−/−^ forebrains as compared to wild-type controls. **F.** Expression of cyclin-dependent kinase inhibitor *Cdkn1a* was tested in different mutant mouse lines. *Cdkn1a* expression was decreased in *Foxg1*-deficient (Foxg1^−/−^) mice as well as in animals where both *Foxg1* and *Tgfbr2* were knocked out (Foxg1;Tgfbr2 dKO). *Cdkn1a* expression was unaffected in mice where *Tgfbr2* was conditionally knocked out in *Foxg1*-expressing cells (Tgfbr2 cKO) and in double knockouts for *Tgfb2* and *Tgfb3* (Tgfb2;Tgfb3 dKO). Results are expressed as Log_2_(fold change) as compared to control wild-type animals (set as 0). **p < 0.01, *p < 0.05; Student's *t*-test; *n* = 3.

We next investigated the transcriptional changes in FOXG1-deficient compared to wild-type CPCs in conditions of active TGFβ-signaling until DIV4. Using microarray technology, we identified 586 differentially expressed genes in FOXG1-deficient compared to wild-type cells using a cutoff of > +1.5 or < −1.5 Log_2_(fold change) (Log_2_FC) and a p-value ≤ 0.05. The majority of the identified genes showed increased expression (Figure 2A; Table S1). Hence, FOXG1 acts mainly as transcriptional repressor. DAVID (Database for Annotation, Visualization and Integrated Discovery) [26, 27] analyses revealed that loss of FOXG1 affects multiple important cellular processes that are involved in brain development (Figure 2B). Loss of *Foxg1* rendered E13.5-derived CPCs responsive to TGFβ-mediated neuronal differentiation (Figure 1H). Therefore, we analyzed whether *Foxg1* deficiency antagonized the TGFβ-pathway not only through association with the FOXO/SMAD4 complex, but also through altered expression of TGFβ- and IGF-signaling pathway members. Compared to wild-type E13.5 forebrains, loss of *Foxg1* increased expression of *Tgfb1* and *Tgfb2* (Figure 2C)*, Foxo1* and *Foxo3* (Figure 2D), and *Igf1, Igf2,* and *Igfbp2* (Figure 2E). We concluded that FOXG1 is not only antagonizing TGFβ- and FOXO-functions on the protein level but also on the transcriptional level.

To further assess the antagonizing function of FOXG1 on SMAD- and FOXO-driven transcription of *Cdkn1a*, we analyzed *Cdkn1a* transcription in different mouse models in E13.5 forebrains. To address the impact of FOXG1 on *Cdkn1a* transcription, we used *Foxg1* knockout mice (Foxg1^−/−^). The TGFBR2 is specifically activated by the TGFβ-ligands, whereas the TGFBR1 is also activated by other ligands of the TGFβ-superfamily. We therefore used TGFBR2-deficient mice to assess the involvement of TGFβ-signaling in *Cdkn1a* transcription. TGFBR2-deficiency was achieved through conditional knockout of *Tgfbr2* using a cre-knockin into the *Foxg1*-gene (Tgfbr2 cKO). Accordingly, these mice express reduced levels of FOXG1 and have impaired TGFBR2-signaling in the same cells. We also generated FOXG1 and TGFBR2 double-deficient mice (Foxg1;Tgfbr2 dKO). Whereas TGFB1 is mainly expressed in the meninges of the forebrain, TGFB2 and TGFB3 ligands are mainly expressed by neural cells [28]. We therefore assessed *Cdkn1a* in TGFB2 and 3 double deficient forebrains (Tgfb2;Tgfb3 dKO), which served as additional model system for impaired TGFβ-signaling. As displayed in Figure 2F, only the complete loss of *Foxg1* increased expression of *Cdkn1a in vivo*. At this developmental stage, loss of TGFBR2 in FOXG1-expressing cells or constitutive lack of TGFB2 and 3 ligands did not seem to be relevant for *Cdkn1a* transcription *in vivo*.

### FOXG1, FOXO1 and SMAD4 form a regulative network to drive their own and *Cdkn1a* expression

**Figure 3:**
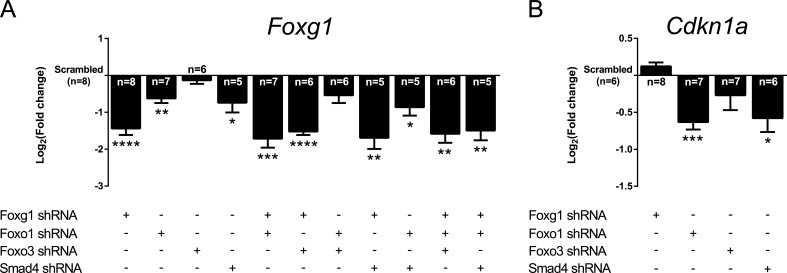
Knockdowns of *Foxg1*, *Foxo1* and *Smad4* affect expression of each other and of *Cdkn1a* **A.**, **B.** E13.5 murine CPCs were infected with shRNA constructs targeting specific genes or scrambled shRNA construct (control). Expression levels of *Foxg1* and *Cdkn1a* were assessed by qRTPCR. **A.** *Foxg1* transcript levels were significantly decreased when shRNA constructs targeted *Foxg1*, *Foxo1*, *Smad4* or their combinations. **B.** *Cdkn1a* expression was significantly decreased upon knockdown of *Foxo1* or *Smad4*, but not upon *Foxo3* or *Foxg1* knockdown. Results are shown as mean of Log_2_(fold change)±SEM in specific shRNA construct condition *vs*. scrambled control (set as 0). ****p < 0.0001, ***p < 0.001, **p < 0.01, *p < 0.05; One-sample *t*-test; replicate numbers indicated in graphics.

FOXG1, FOXO1, FOXO3 and SMAD4 form a network implicated in controlling *Cdkn1a* expression. In addition, FOXG1 interfered with transcription of *Foxo1* and *Foxo3 in vivo* (Figure 2D). We therefore evaluated transcriptional changes of this network on its own members and on *Cdkn1a in vitro* using shRNA-mediated knockdown of single components and their combinations. Knockdown of *Foxg1* led to a moderate decrease of its own expression by approximately 63% (log_2_FC = −1.44; Figure 3A). This is little less than the *Foxg1* expression that we observed in heterozygote animals (Figure S1). *Foxg1* transcription was also decreased after knockdown of *Foxo1* and of *Smad4*, either alone or in combination. In contrast, the expression of *Foxo1, Foxo3* and *Smad4* was exclusively affected when shRNAs against their own mRNAs were used (Figure S2). Thus, although loss of *Foxg1 in vivo* indicated that FOXG1 is implicated in transcriptional control of *Foxo1* and *Foxo3*, the level of suppression of FOXG1 using the shRNA (63%) was not sufficient to induce *Foxo1* and *Foxo3* transcription *in vitro*.

We next assessed the expression of *Cdkn1a* in this *in vitro* setup. We observed that shRNA-mediated *Foxg1* knockdown did not cause an increase in *Cdkn1a* expression (Figure 3B). We concluded that even low expression of *Foxg1* efficiently blocked the transcription of this cell cycle inhibitor. This conclusion is supported by the *in vivo* data, which showed that loss of one *Foxg1* allele is not sufficient to increase *Cdkn1a* levels *in vivo* in Tgfbr2 cKO, whereas loss of both *Foxg1* alleles strongly increases *Cdkn1a* levels (Figure 2F). Knockdown of *Foxo1* and *Smad4* suppressed *Cdkn1a* levels. In contrast, knockdown of *Foxo3* did not result in significant changes of *Cdkn1a* transcription (Figure 3B).

We concluded that FOXG1 suppresses the expression of *Foxo1*, *Foxo3* and *Cdkn1a*. FOXO1 in turn increases not only the expression of *Cdkn1a* but also that of its repressor *Foxg1*. *Smad4* also increases *Cdkn1a* and *Foxg1* transcription *in vitro*. Thus, aside from forming protein complexes with FOXO and SMAD4, FOXG1 interferes with the transcription of *Tgfβ* and *Foxo* genes that oppose mitosis of cortical progenitors.

### IGF-signaling drives *Cdkn1a* expression through FOXO1

**Figure 4:**
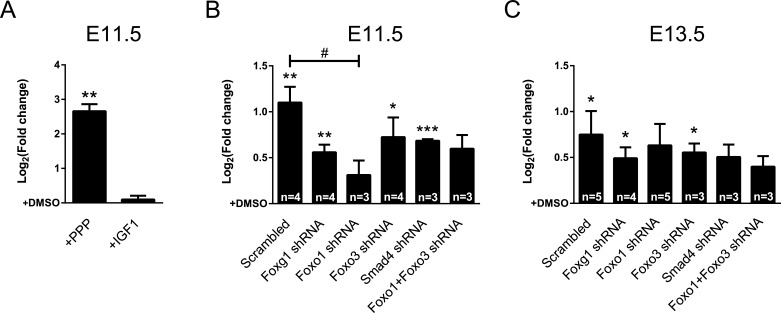
IGF1-pathway affects *Cdkn1a* expression through FOXO1 **A.** *Cdkn1a* expression in E11.5 CPCswas significantly increased after blocking intracellular IGF1-signaling with PPP. Treatment with IGF1 did not affect *Cdkn1a* expression. **B.**, **C.** *Cdkn1a* expression was assessed in E11.5 (B) and E13.5 (C) CPCs after knocking down the expression of *Foxg1*, *Foxo1*, *Foxo3*, *Smad4* or *Foxo1+Foxo3* while blocking IGF1-signaling by PPP treatment. (B) Expression of *Cdkn1a* increased in E11.5 CPCs upon treatment with PPP except when *Foxo1* expression was knocked down. (C) *Cdkn1a* expression in E13.5 CPCs was not significantly raised upon PPP treatment when either *Foxo1* or *Smad4* expression was knocked down. Results are shown as mean of Log_2_(fold change)±SEM of each PPP-treated condition *vs*. relative DMSO-treated control (set as 0). ***p < 0.001, **p < 0.01, *p < 0.05; One-sample *t*-test (*vs*. DMSO-treated control). #p < 0.05; One-way ANOVA - Dunnet's post-test (*vs*. Scrambled control). Replicate number in A: *n* = 4.

IGF-signaling activates PI3K, which subsequently leads to phosphorylation of FOXO proteins and exclusion from the nucleus [12, 29, 30]. As our data showed that deficiency of FOXG1 increased expression of IGF-pathway members (Figure 2E), we analyzed the effects of IGF-signaling on *Cdkn1a* expression in the context of the FOXG1/FOXO1/FOXO3/SMAD4 transcriptional network. We have recently shown that IGF-signaling is highly active in CPCs that derive from early developmental time points (E11.5 and E13.5), whereas it does not have large impact at later stages (E16.5) [2]. Hence, we hypothesized that IGF-signaling is involved in *Cdkn1a* expression in E11.5 and E13.5 CPCs. We isolated CPCs from E11.5 mouse brains, treated them with Picropodophyllin (PPP), IGF1 or DMSO. Using qRTPCR, we observed that *Cdkn1a* expression increased in E11.5-derived CPCs when IGF1-signaling was blocked with PPP (Figure 4A). This result is in accordance with IGF-dependent activation of AKT-mediated phosphorylation of FOXO proteins that translocates FOXOs into the cytoplasm and interferes with their activating effects on transcription. It also reflects that IGF-signaling mediates proliferation of CPCs. Treatment of E11.5-derived CPCs with IGF1 did not result in transcriptional repression of *Cdkn1a* (Figure 4A). This is probably because of high expression levels of IGF1 at these early developmental stages [2] that might be sufficient to fully activate the pathway.

Next, we analyzed whether IGF1-signaling interfered with *Cdkn1a* transcription by impinging on FOXO1 and/or FOXO3. The shRNA knockdown experiments (Figure 3B) suggested that FOXO1 activates *Cdkn1a* expression, which might be prevented by IGF-mediated FOXO1 phosphorylation (Figure 4A). Hence, interference with FOXO1 expression should prevent transcriptional activation of *Cdkn1a* after inhibition of IGF1-signaling. In contrast, reduced FOXO3 expression might not alter *Cdkn1a* transcript levels when IGF1-signaling is inhibited. To show this, we infected E11.5-derived CPCs with lentiviral shRNAs against specific members of the FOXG1/FOXO1/FOXO3/SMAD4 transcriptional network and subsequently blocked IGF1-signaling with PPP. Blocked IGF1-signaling induced *Cdkn1a* transcription in nearly all conditions compared to respective DMSO controls, except in conditions with a knockdown of *Foxo1* (Figure 4B). In addition, compared to the scrambled control, knockdown of *Foxo1* was the only condition, which resulted in significantly decreased expression of *Cdkn1a*. This finding further corroborated that FOXO1 but not FOXO3 is important to activate *Cdkn1a* transcription. Next, we assessed whether these transcriptional changes were also observed in E13.5 CPCs. Although induction of *Cdkn1a* transcription was generally less pronounced compared to E11.5-derived cells, IGF-signaling mainly affected FOXO1-mediated transcriptional activation of *Cdkn1a*. In accordance with our observations in E11.5-derived CPCs, interference with *Foxo1* expression in E13.5-derived cells was one condition in which *Cdkn1a* was not significantly induced after PPP treatment (Figure 4C). Interference with *Smad4* reduced *Cdkn1a* expression after PPP treatment as well, indicating a growing influence of the TGFβ pathway on *Cdkn1a* transcription during development. Again, interference with *Foxo3* expression did not impair *Cdkn1a* expression after PPP treatment. We did not observe changes in localization of FOXO3 upon PPP or IGF1 treatment (Figure S3). This further suggests that FOXO3 might not be relevant in IGF1-mediated control of *Cdkn1a* expression. Hence, we concluded that expression of *Cdkn1a* is activated by FOXO1, and not by FOXO3 in E11.5 and E13.5 cells.

### TGFβ-signaling is not sufficient to induce differentiation of *Calb2*-expressing Cajal-Retzius cells

**Figure 5:**
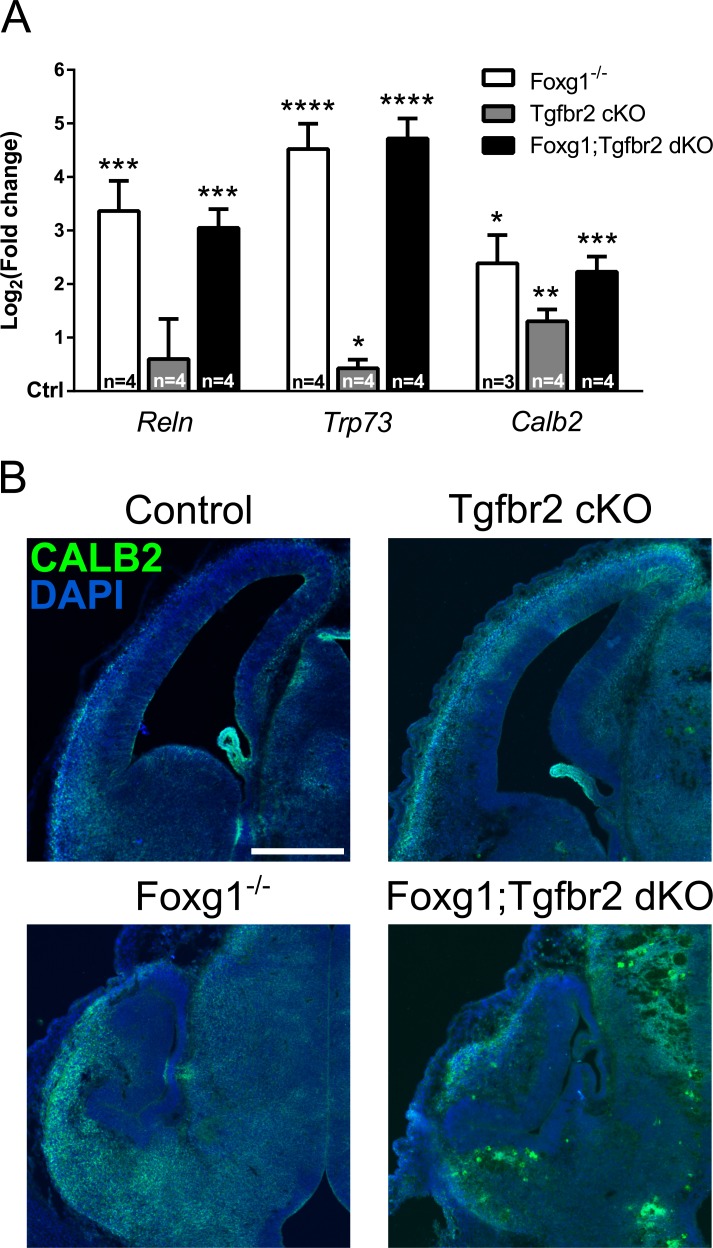
Evaluation of FOXG1/TGFβ-pathway crosstalk in specification of Cajal-Retzius (CR) cells **A.** qRTPCR-based analysis of the expression of CR cell markers in E13.5 Foxg1^−/−^, Tgfbr2 cKO and Foxg1;Tgfbr2 dKO mice. Transcriptional expression of *Reln*, *Trp73* and *Calb2* was significantly increased in all models lacking *Foxg1* expression. *Reln* expression was not affected by loss of *Tgfbr2. Trp73* and *Calb2* transcripts were mildly increased in Tgfbr2 cKOs. **B.** Immunohistochemical analysis of brain sections from E13.5 Foxg1^−/−^, Tgfbr2 cKO, Foxg1;Tgfbr2 dKO as well as corresponding controls, showed that CALB2 expression is increased upon loss of *Foxg1* expression. Consistent with the qRTPCR results, the amount of CALB2-positive cells was not decreased in Tgfbr2 cKO mice. qRTPCR results are shown as mean of Log_2_(fold change)±SEM of each condition *vs*. relative control (set as 0). ****p < 0.0001, ***p < 0.001, **p < 0.01, *p < 0.05; Student's *t*-test; n≥3. Scale bar: 250 μm.

The FOXG1/FOXO1/FOXO3/SMAD4-network does not only control cell cycle exit, but also regulates differentiation of CR cells. TRP73-expressing CR cells derive from the cortical hem, which does not express FOXG1. Another CR-producing region is the pallial-subpallial boundary (PSB), in which progenitors express FOXG1 and generate CALB2-positive CR neurons. In the cortical hem, TGFβ-signaling is involved in the generation of TRP73-positive CR cells, because FOXG1 is not present in this region and is not antagonizing CR cell generation [19]. We explored whether TGFβ increased CR cell differentiation in the FOXG1-expressing PSB in conditions of reduced FOXG1 levels. We assessed expression of *Reln*, *Trp73* and *Calb2* in Foxg1^−/−^, Tgfbr2 cKO and Foxg1;Tgfbr2 dKO forebrains. *Foxg1*-deficient mice expressed increased levels of *Reln*, which indicates general CR cell overproduction. The Tgfbr2 cKO mice did not show increased *Reln* expression. *Trp73* was strongly increased in both *Foxg1*-deficient mouse models, but only slightly in Tgfbr2 cKO (Figure 5A). Expression of *Calb2* increased in all three mouse models as compared to control animals. We concluded that FOXG1 expression is not antagonizing TGFβ activated differentiation of *Calb2*-expressing CR from the PSB. Otherwise, expression of *Calb2* would be reduced in forebrains that are deficient for both, TGFβ-signaling and FOXG1. This result was also confirmed by immunohistochemistry stainings of CALB2 in the different mouse lines (Figure 5B).

### Overexpression of *Cdkn1a* or interference with FOXO1 or FOXO3 does not alter neuronal differentiation

**Figure 6:**
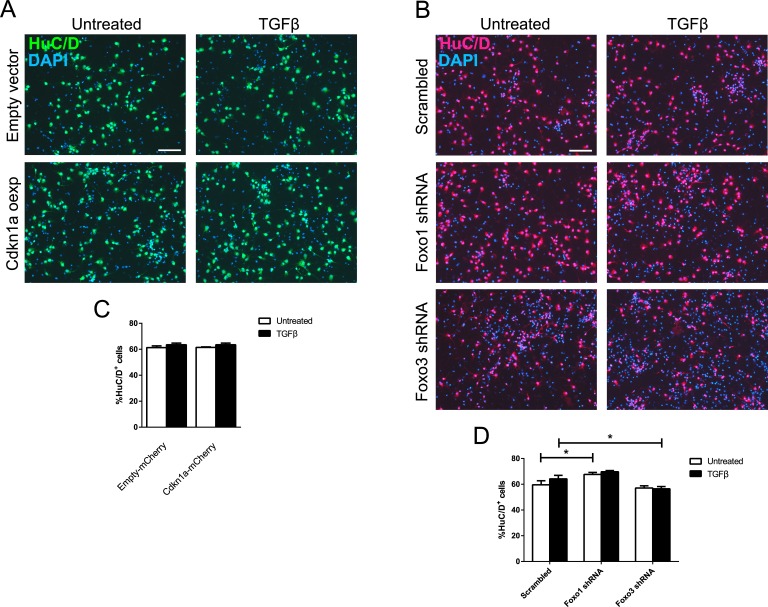
Neuronal differentiation at E13.5 is unaffected by *Cdkn1a* overexpression or loss of *Foxo1* and *Foxo3* **A.**, **C.** Immunocytochemical analysis of HuC/D expression (A) showed that overexpression of *Cdkn1a* is not sufficient to induce neuronal differentiation in E13.5 CPCs. The percentage of HuC/D^+^ cells after *Cdkn1a* overexpression with or without TGFB1 stimulation (6 days) did not change significantly compared to E13.5 CPCs infected with empty vector (C). **B.**, **D.** Immunocytochemical staining for HuC/D performed on E13.5 CPCs infected with shRNA constructs targeting *Foxo1* or *Foxo3*, either treated with TGFB1 for 6 DIV or left untreated. Knockdown of *Foxo1* led to a mild but significant increase in untreated HuC/D^+^ cells, while knockdown of *Foxo3* expression decreased the amount of TGFβ-treated HuC/D^+^ cells. *p < 0.05; One-way ANOVA - Šidák's post-test for comparison of specific pairs; *n* = 3. Scale bar: 100 μm.

We further investigated whether overexpression of *Cdkn1a* alone would be sufficient to increase neuronal differentiation. We infected E13.5-derived CPCs with a lentiviral CDKN1A overexpression construct and stained for the neuronal marker HuC/D. Quantification of HuC/D-positive CDKN1A-overexpressing cells compared to cells carrying the empty vector did not reveal increased neuronal differentiation. This was independent of TGFβ stimulation (Figure 6A, 6C). To assess whether FOXO1 and FOXO3 increased neuronal differentiation, we interfered with their expression using shRNAs in E13.5-derived CPCs and determined the number of HuC/D-expressing neurons. We did not observe a striking influence of either FOXO protein on the generation of neurons. Only mild effects on neuronal differentiation were uncovered that opposed each other (Figure 6B, 6D). Whereas interference with FOXO1 led to a small but significant increase in the number of neurons in untreated conditions, interference with FOXO3 led to a slight decrease in HuC/D-positive neurons after stimulation with TGFβ, indicating a mild differentiating effect of FOXO3. We concluded that FOXO1 and FOXO3 might have opposing effects on neuronal differentiation and that FOXO1 and FOXO3 have non-redundant functions, which were also revealed by differences in the transcriptional control of *Cdkn1a* and *Foxg1*. These data also indicated that FOXO1 does not activate downstream genes that increase neuronal differentiation at E13.5.

### FOXG1 activates *Kcnh3* transcription

**Figure 7:**
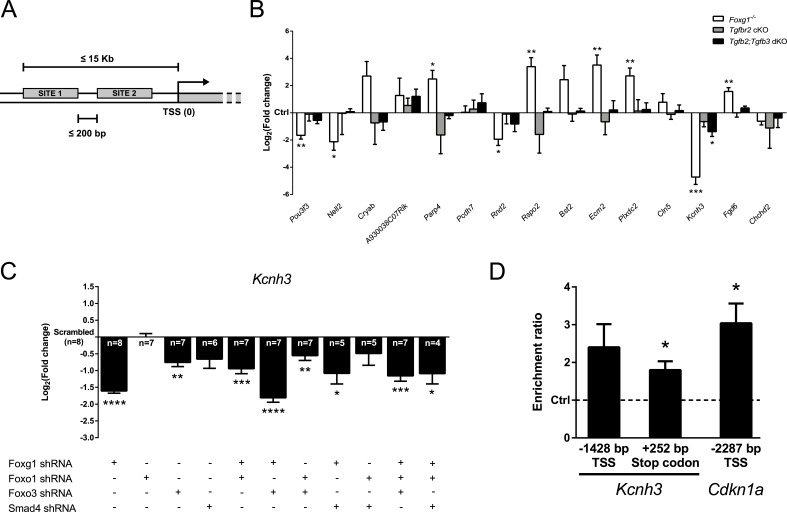
Identification of novel candidate genes regulated through FOXG1/SMAD crosstalk **A.** Whole mouse genome was screened for genes possessing both Forkhead box- (GTAAACAA) and SMAD4-specific (AGAC) consensus DNA-binding sites in a range of 15 Kb before the TSS and placed not farther than 200 bp from each other. Selected candidate genes fulfilled these criteria and were regulated in our microarray analysis. **B.** Transcriptional expression of candidate genes was assessed by qRTPCR using E13.5 telencephalic hemispheres from Foxg1^−/−^, *Tgfbr2* cKO and *Tgfb2;Tgfb3* dKO mice. Results are expressed as Log_2_(fold change)±SEM of target gene expression in mutants as compared to respective controls (Ctrl, set as 0). *Kcnh3* was found to be regulated in both Foxg1^−/−^ and Tgfb2;Tgfb3 dKO mice and was thus selected for further analyses. ***p < 0.001, **p < 0.01, *p < 0.05; Student's *t*-test; *n* = 3. **C.** *Kcnh3* expression in wild-type E13.5 CPCs infected with shRNA constructs targeting *Foxg1*, *Foxo* genes and *Smad4* decreased when either *Foxg1* or *Foxo3* were knocked down. Results are shown as mean of Log_2_(fold change)±SEM in specific shRNA construct condition *vs*. scrambled control (set as 0). ****p < 0.0001, ***p < 0.001, **p < 0.01, *p < 0.05; One-sample *t*-test; replicate numbers indicated on graphics. **D.** Potential binding of FOXG1 at specific sites upstream of *Kcnh3* as well as at the 3′-UTR was investigated using ChIP. FOXG1 was enriched in 3′-UTR region (252-287 bp downstream from the termination codon; reference NM_010601.3). Significant FOXG1 binding also occurs on the *Cdkn1a* locus (2287-2252 bp upstream of TSS; reference NM_007669.4) at the FOX-binding site described by Seoane et al. [7]. Results are shown as a ratio between FOXG1 enrichment at a specific site and its enrichment in a region devoid of FOX binding sites, which was used as a negative control (set as 1 and shown by a dashed line). *p < 0.05; One sample *t*-test; *n* = 5.

We further analyzed our microarray data to assess which genes other than *Cdkn1a* were regulated by FOXG1 in a context of active TGFβ-signaling. Comparison between expression profiles of TGFβ-treated Foxg1^−/−^ and wild-type E13.5 CPCs showed differential expression of many genes involved in a great variety of biological processes (Figure 2A, 2B; Table S1). Such processes spanned from neuronal differentiation to apoptosis, from cell migration and adhesion to angiogenesis. To identify genes that are regulated through a FOXG1/TGFβ crosstalk, we first analyzed the whole mouse genome by searching for gene promoters that contained both FOX (GTAAACAA) and SMAD4 (AGAC) DNA-binding sites using SiteSearch software [31]. For this genome-wide screen, we analyzed DNA regions 15 Kb upstream of all transcription start sites (TSS), and added the constraint that both FOX and SMAD4 binding sites must be located within 200 bp of each other (Figure 7A). The resulting gene list was compared with the microarray results and shortlisted candidates were validated through qRTPCR analysis. We used RNA from cerebral cortex of E13.5 Foxg1^−/−^, Tgfbr2 cKO and Tgfb2;Tgfb3 dKO mice, respectively. Many candidates were regulated in Foxg1^−/−^ mice, confirming the microarray results (Figure 7B; Table S1). Out of all short-listed genes only *Kcnh3*, a gene encoding for a member of the *ether-à-go-go* family of voltage gated K^+^ channels [32], was transcriptionally decreased in both Foxg1^−/−^ and Tgfb2;Tgfb3 dKO. However, Tgfbr2 cKO mice did not reveal significant changes in the expression of *Kcnh3*. Thus, although FOX and SMAD4 DNA-binding sites were identified upstream of the *Kcnh3* TSS, the influence of TGFβ-signaling on *Kcnh3* expression was less compared to FOXG1. In contrast to its repressive function in *Cdkn1a* transcription, FOXG1 activated *Kcnh3* transcription. We next assessed whether further members of the FOXG1/FOXO1/FOXO3/SMAD4 transcriptional network were also involved in *Kcnh3* expression. We used lentiviral shRNAs to interfere with the expression of individual members and combinations of them *in vitro*. Corroborating our *in vivo* results, *Kcnh3* was strongly decreased in all conditions of FOXG1-deficiency (Figure 7C). Whereas FOXO1 was not involved in transcriptional regulation of *Kcnh3*, loss of FOXO3 impaired *Kcnh3* expression. Interference with SMAD4 did not significantly change *Kcnh3* expression, supporting our *in vivo* results that indicated no significant contribution of TGFBR2-mediated signaling to *Kcnh3* expression. We concluded that *Kcnh3* expression is increased by FOXG1 and FOXO3, but is independent of FOXO1 and TGFβ-signaling.

For a more detailed analysis of the *Kcnh3* promoter, we used the ConTra v2 software [33], which allows analyses of individual genes as compared to the genome-wide approach of SiteSearch. ConTra v2 matrix-based sequence analysis of conserved motifs was performed within the promoter region and in the 3′-UTR of *Kcnh3.* The promoter analysis was restricted to 1500 bp upstream of the TSS. Using this method, we identified the closely located and conserved FOX- and SMAD4-binding sites 1428-1397 bp upstream of the *Kcnh3* TSS (reference NM_010601.3). As our expression data did not reveal TGFβ-dependent transcription of *Kcnh3*, we also analyzed the *Kcnh3* gene locus for isolated FOX-binding sites independent of SMAD4-target sequences. We identified an additional conserved FOX target sequence in the 3′-UTR region 252-287 bp downstream of the termination codon of *Kcnh3*. The study by Seoane et al. [7] did not report whether FOXG1 is localized at the chromatin of the *Cdkn1a* gene. Thus, we aimed to reveal whether FOXG1 is present at the putative binding sites in the *Cdkn1a* and *Kcnh3* genes. We employed chromatin immunoprecipitation (ChIP) of FOXG1 on chromatin isolated from dorsal telencephalic CPCs derived from E13.5 wild-type mice. Enrichment at the specific sites was compared to an intergenic region devoid of putative FOX protein DNA-binding sequences (Figure 7D). We revealed strong and significant enrichment of FOXG1 at the forkhead binding site in the *Cdkn1a* gene that is located near the SMAD4-binding sites 2287-2251 bp upstream of the TSS (reference NM_007669.4). Hence, *Cdkn1a* repression by FOXG1 probably includes its presence on the chromatin. We also detected significant enrichment of FOXG1 at the *Kcnh3* 3′-UTR site, whereas the enrichment at the upstream candidate sequence showed a trend but was not significant (Figure 7D). We therefore concluded that FOXG1 exerts transcriptional activation or repression either directly or indirectly associated with the chromatin.

### *Kcnh3* is mainly expressed in lateral pallium, lateral entorhinal cortex and hippocampus during development

As the expression pattern of *Kcnh3* during embryonic development has not yet been described, we performed *in situ* hybridization (ISH) for *Kcnh3* on wild-type murine brain sections at different developmental stages (E11.5, E13.5 and E16.5).

**Figure 8:**
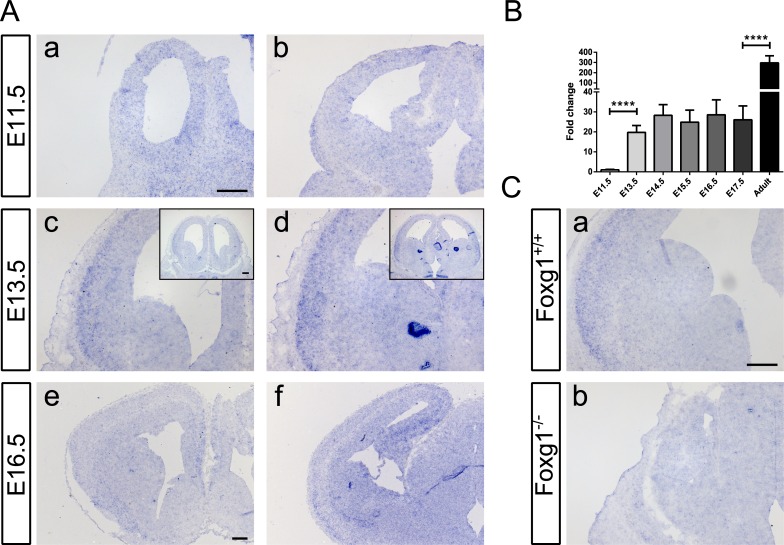
Characterization of spatial and temporal expression of *Kcnh3* in the developing murine forebrain **A.** *Kcnh3*-expressing regions were identified by *in situ* hybridization on sections from E11.5 (Aa-b), E13.5 (Ac-d) and E16.5 (Ae-f) murine forebrains. While no specific staining could be detected at E11.5 (Aa-b), at E13.5 weak to mild expression was detectable in the regions corresponding to the rostral lateral pallium (Ac) to the developing lateral entorhinal cortex (Ad). At E16.5, *Kcnh3* expression took place in the same regions as at E13.5 (Ae-f), as well as in the developing hippocampus (Af). **B.** qRTPCR analysis showed transcriptional levels of *Kcnh3* in the forebrain at different developmental stages (E11.5, E13.5, E14.5, E15.5, E16.5, E17.5 and adult). *Kcnh3* expression increased between E11.5 and E13.5, remained stationary until E17.5, and increased at the adult stage. Values are expressed as relative fold change in comparison to E11.5 (indicated as 1). ****p < 0.0001; One-way ANOVA – Šidák's post-test comparing consecutive developmental stages; *n* = 3. **C.** *Kcnh3* expression in forebrains of Foxg1^−/−^ (Cb) as compared to wild-type (Ca) animals. *Kcnh3* was undetectable in Foxg1^−/−^. Experimental replicates: *n* = 3 for all in situ hybridization experiments, except for Foxg1^−/−^ (*n* = 2). Scale bar: 200 μm.

No specific staining was detected at E11.5 (Figure 8Aa-b). At E13.5, a weak to mild expression occurred from the region corresponding to the rostral lateral pallium (Figure 8Ac) to the developing lateral entorhinal cortex (Figure 8Ad). At E16.5, the detection of the *Kcnh3* transcript extended to the developing hippocampus (Figure 8Ae-f). qRTPCR analysis of *Kcnh3* transcript levels in cerebral hemispheres at different developmental stages were consistent with the results obtained by ISH. The expression increased from nearly undetectable levels (E11.5) to moderate levels (E13.5 to E17.5), finally reaching a high level of expression at the adult stage (Figure 8B).

ISH performed on sections from Foxg1^−/−^ animals (Figure 8Cb) did not show any noticeable *Kcnh3* signal when compared to sections from wild-type controls (Figure 8Ca). These observations confirmed the qRTPCR results of *Kcnh3* expression in Foxg1^−/−^ animals (Figure 7B), and in CPCs after shRNA-mediated *Foxg1* knockdown (Figure 7C), in which *Kcnh3* levels were also decreased compared to controls. We concluded that *Kcnh3* is a direct target of FOXG1 and that its expression is mainly confined to mature neurons, the generation of which is impaired in *Foxg1*-deficient mice.

## DISCUSSION

Seoane et al. [7] established the FOXG1/FOXO/SMAD model of interacting proteins that are involved in controlling proliferation of neuroepithelial cells by acting on the *Cdkn1a* promoter. In the pathological case, this network is important in keeping glioblastoma cells in a proliferative state and in conferring escape from TGFβ-mediated cytostasis. We discovered that FOXG1 expression impaired not only FOXO/SMAD-mediated cell cycle exit, but also TGFβ-induced neuronal differentiation in a dose dependent manner at the developmental stage E13.5. Our *in vitro* data indicate that FOXO1 is an activator of *Cdkn1a* expression from E11.5 onwards, and that it is probably assisted by SMAD4 from E13.5 onwards (Figures 3 and 4). Our *in vivo* data do not indicate that TGFβ-signaling *via* TGFBR2 or the neural ligands TGFB2 and 3 are essential regulators of *Cdkn1a* transcription. The reason might be that either FOXO1/SMAD4-mediated *Cdkn1a* expression is only transient during the period when a progenitor leaves the cell cycle and enters neuronal differentiation. Alternatively, as our *in vitro* data indicate a robust SMAD4-dependent transcription of *Cdkn1a*, SMAD-signals might be under control of alternative pathways, such as BMP or Activin. In addition, on the basis of our data we cannot rule out that other compensatory mechanisms, such as activation of TGFβ-signaling in neural cells by increased levels of TGFB1, might cloud our analyses of the full impact of TGFβ-signaling on *Cdkn1a* expression *in vivo*.

**Figure 9:**
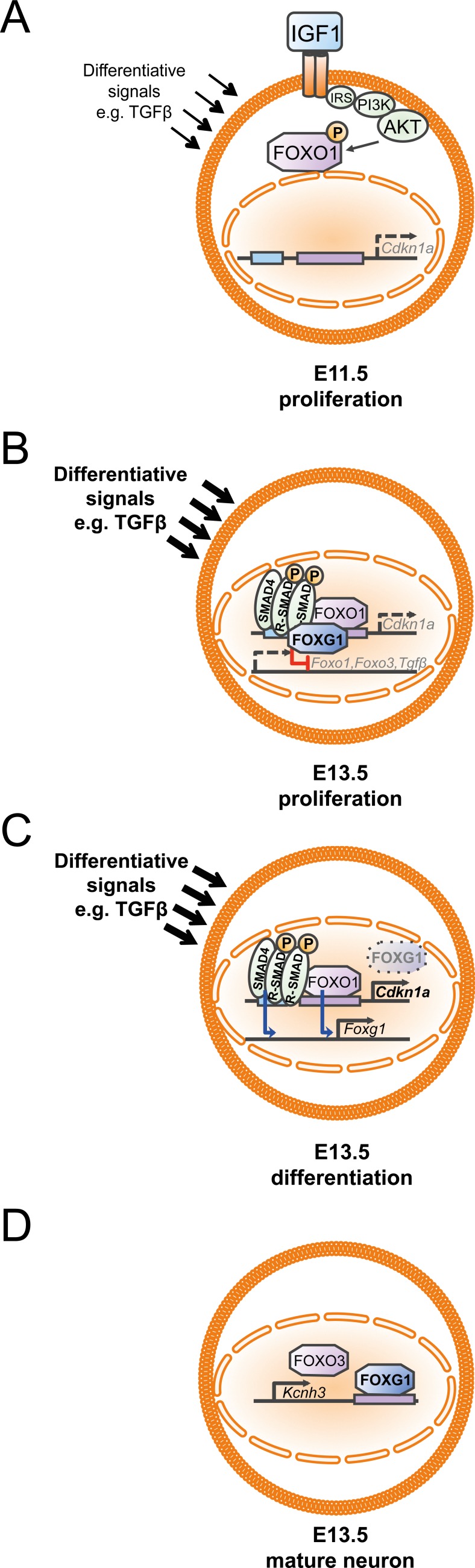
Developmental dynamics of the FOXG1/FOXO/SMAD4 network in the context of cortical progenitor proliferation and differentiation **A.** At E11.5 proliferation is dominant and expression of *Cdkn1a* through FOXO1 protein is prevented by IGF1-mediated activation of the AKT-signaling pathway that keeps FOXO1 in the cytoplasm due to phosphorylation. **B.** As development progresses, progenitors are exposed to increasing amounts of differentiating signals. To prevent exit from mitosis FOXG1 associates with FOXO1 and SMAD4 complexes at the *Cdkn1a* promoter and thereby FOXG1 prevents cell cycle exit. At the same time, FOXG1 represses the expression of *Foxo1*, *Foxo3* and *Tgfβ*. **C.** In differentiating progenitors, transient downregulation of FOXG1 allows differentiating signals such as TGFβ to drive neuronal differentiation, which follows FOXO1/SMAD4 mediated transcription of *Cdkn1a*. To allow differentiated cells to integrate into the cortical plate, FOXO1/SMAD4 proteins drive *Foxg1* expression. **D.** FOXG1 binds to the 3′-UTR of *Kcnh3* and activates expression of *Kcnh3* in mature neurons. FOXO3 also drives *Kcnh3* expression.

The FOXG1/FOXO/SMAD network was further characterized in the context of differentiation of Cajal-Retzius neurons [19]. Our data do not support the hypothesis that FOXG1 antagonizes a putative involvement of TGFβ-signals in the generation of CALB2-positive CR cells from the PSB, as we revealed similar expression of CALB2 in Foxg1-/Tgfbr2-double deficient mice compared to FOXG1-deficient mice. However, we cannot rule out that SMAD4-dependent transcriptional regulation might promote CR differentiation, and that activation of SMAD4 occurs in response to BMP or Activin signals, independent of TGFβ ligands and receptors. Our additions to the existing model [7] show that the FOXG1/FOXO/SMAD transcriptional network acts in different complexes to confer different cellular responses depending on the developmental time point (Figure 9). In early developmental stages, such as E11.5, progenitors mainly proliferate. Under these conditions, IGF1-signaling secures the proliferating status by preventing FOXO1 translocation to the nucleus to drive *Cdkn1a* expression (Figure 9A, Figure 4, [2]). At E13.5 most progenitors still divide, but neuronal differentiation becomes increasingly important, and both proliferation and differentiation coexist. Thus, with progressing development, the progenitors are exposed to signals that increase neuronal differentiation although a subset of progenitors has to maintain the capacity for mitosis. As our data suggest, differentiating signals include TGFβ. FOXG1-expressing progenitors stay in mitosis (Figure 9B) as FOXG1 associates with FOXO1 and SMAD complexes [7], blocks transcription of *Tgfβ, Foxo1, Foxo3,* and *Cdkn1a* (Figures 2, 3), binds to the *Cdkn1a* promoter (Figure 7) and hampers neuronal differentiation upon a TGFβ-stimulus (Figure 1). Loss of FOXG1 (Figure 9C) renders progenitors capable to express *Cdkn1a* through FOXO1 and SMAD4 (Figures 2, 3) and to enter neuronal differentiation (Figure 1). Along this line, Miyoshi and Fishell [18] reported that transient downregulation of FOXG1 is needed in pyramidal neuron precursors to enter into the cortical plate. We show that FOXO1 and SMAD4 are strong candidates to drive the re-expression of *Foxg1* in differentiating pyramidal neurons (Figure 3). Our finding that FOXG1 increases expression of *Kcnh3* extends the view that FOXG1 has important functions in mature neurons beyond integration of pyramidal neurons into the cortical plate (Figure 9D).

Our data show that FOXG1 acts antagonistic to FOXO1 but along the same line as FOXO3. This reflects first of all non-redundant functions of the FOXO proteins, as FOXG1 opposes FOXO1-mediated transcription of *Cdkn1a* but drives *Kcnh3* expression, which we also observed for FOXO3. It is possible that the position of the FOX-binding site determines the transcriptional readout. If the binding site is present at the 5′ end it might be antagonistic (*Cdkn1a*), while presence at the 3′ end might be synergistic (*Kcnh3*). Our observation that FOXG1 increases *Kcnh3* expression may also bear clinical relevance, as knockout mice for *Kcnh3* show improved cognitive performance, enhanced spatial working memory and enhanced latent learning memory [32]. In addition, *Kcnh3*-deficient mice show signs of persistent neuronal hyperexcitability including frequent interictal spiking and spontaneous seizures [34]. Early-onset seizures are also a specific feature of the pathology of atypical Rett syndrome, which is caused by *FOXG1* haploinsufficiency in humans (FOXG1 syndrome, OMIM#164874) [35–42]. It is tempting to speculate that reduced expression of KCNH3 is involved in the pathology of the FOXG1-syndrome in humans.

## CONCLUSIONS

These data show that the FOXG1/FOXO/SMAD transcription factor network balances proliferation and differentiation in cortical progenitors. Our data strongly suggest that the network is dynamically involved in the control of cell cycle exit and neuronal differentiation. The network components are used differently while development progresses. *Cdkn1a* expression underlies IGF1-controlled interference of nuclear translocation of FOXO1 at E11.5, when neither TGFβ nor FOXG1 are strongly expressed. At later developmental stages *Cdkn1a* expression is prevented through FOXG1-mediated interference with FOXO1/SMAD4. FOXO1/SMAD4 complexes induce *Foxg1* expression, which makes them candidates for resuming FOXG1 expression in differentiating cortical neurons, which have left mitosis after transient downregulation of FOXG1. FOXG1 and FOXO3 are of relevance to control expression of the seizure-related *Kcnh3.* FOXG1-mediated expression of ion channels could be relevant to understand the pathology of the FOXG1 syndrome, an autism spectrum disorder.

## MATERIALS AND METHODS

### Mouse strains and genotyping

Foxg1^−/−^, Foxg1^cre/+^;Tgfbr2^flox/flox^ (Tgfbr2 cKO), Foxg1^cre/cre^;Tgfbr2^flox/flox^ (Foxg1;Tgfbr2 dKO) and Tgfb2^−/−^;Tgfb3^−/−^ (Tgfb2;Tgfb3 dKO) were obtained and genotyped as previously described [43–46]. Bl6N and NMRI mice were obtained from Charles River and Janvier. The animal welfare committee of the University of Freiburg and local authorities approved all animal experiments (registered license X11/09S and X14/04H). For genotyping, tails were lysed in QuickExtract DNA Extract Solution (Biozym Scientific GmbH, Hessisch Oldendorf, Germany) according to manufacturer's instructions. PCR was performed using Taq polymerase (Go-Taq, Promega, Fitchburg, WI, USA) and according to instructions the following primers (Invitrogen, Carlsbad, CA, USA; Sigma, St. Louis, MO, USA) were used:

Bf1_F25 GCCGCCCCCCGACGCCTGGGTGATG

Bf1_R159 TGGTGTGGTGATGATGATGATGGTGATGCTG

Bf1_Rcre ATAATCGCGAACATCTTCAGGTTCTGGGGG

LaLox ACTTCTGCAAGAGGTCCCCT

8wa TAAACAAGGTCCGGAGCCCA

b2-3 AACTCCATAGATATGGGGATGC

b2_neo GCCGAGAAAGTATCCATCAT

b2-5 AATGTGCAGGATAATTGCTGC

### Primary cultures of mouse embryonic cortical cells

Cortical cells were isolated from embryonic mice at various time points (E11.5, E13.5, E16.5), dissected in Hanks' Balanced Salt Solution (HBSS, PAA, Cölbe, Germany) and dissociated by trypsinization in 0.25% Trypsin-ethylenediaminetetracetic acid (PAA) at 37°C for 7 min. Half a volume of Fetal Bovine Serum (FBS, Life Technologies) and one volume of Neurobasal (NB) complete medium supplemented with B27 (both from Life Technologies), apo-transferrin (5 μg/ml, Sigma), superoxide-dismutase (0.8 μg/ml, Sigma), L-glutamine (0.5 mM, Life Technologies), PSN (Life Technologies), glutathione (1 μg/ml, Sigma) were added. Cells were triturated. DNase treatment (Roche, Basel, Switzerland) was performed to help the trituration process (10 μg DNAse per ml of trypsin). Cells were plated on poly-ornithine (0.1 mg/ml, Sigma, München, Germany) and laminin (1 μg/ml, Sigma) coated dishes at a density of 100000 cells/cm^2^. The day of the plating was considered DIV0. Cells were harvested on different days ranging from DIV4 to DIV9. For the possible treatment schemes, based on the performed experiments, see Figure S4. For immunocytochemistry, TGFB1 (5 ng/ml) was applied at DIV2 or DIV3, according to experimental need.

### Production of lentiviral particles

Lentiviral particles were produced and their titer determined as previously described [45]. Briefly, HEK293T cells were transfected with Mirus TransIT-293 Transfection Reagent (Mirus Bio LLC, Madison, WI, USA) following manufacturer's instructions. The following plasmid concentrations were used for one 10cm dish: 2.4 μg psPax2; 2.4 μg pMD.2-VSVG; 4.8 μg Vector plasmid. Medium was harvested after 72 h post-transfection, pre-cleared by centrifugation and filtered through a 0.45 μm filter. The viral particles were pelleted by ultracentrifugation (25000 rpm for 2 h at 4°C). The viral pellet was resuspended in NB complete medium. Titer was determined by a qRTPCR-based approach.

### Infection with lentiviral particles

Cells were infected on DIV1 with 1.25 infecting units (IFU)/cell (shRNA-mediated knockdown) or 10 IFU/cell (*Cdkn1a* overexpression). The following constructs were used for shRNA-mediated knockdown: shFoxg1 TRCN0000081746 (CCTGACGCTCAATGGCATCTA), shFoxo1 TRCN0000234399 (TGGAAACCAGCCAGCTA TAAA), shFoxo3 TRCN0000312843 (CAGCCGTGCC TTGTCAAATTC) in pLKO.1-puro-CMV-tGFP backbone (all purchased from Sigma). The coding region of *Cdkn1a* (forward primer: GGAATTCCCACCATGTCCAATCCTGGTGATGTC; reverse primer: CGCGGATCCGCGGGGTTTTCTC TTGCAGAAGAC) was cloned into a pLenti-III-2A-mCherry-nopuro backbone (Applied Biological Materials Inc., Richmond, BC, Canada) for overexpression. Selection of successfully infected cells was performed 72-90 h post-infection (DIV4) by changing the medium to NB complete medium with 0.3 μg/ml puromycin (Sigma) for 3 days. For harvesting schedule see Figure S4.

### RNA isolation, reverse transcription, and quantitative real-time PCR (qPCR) and qRTPCR data analysis

Total RNA was isolated from the harvested cells and frozen tissue using RNeasy mini kit (Qiagen, Germany) according to the manufacturer's instructions including DNA digestion. One μg of total RNA was reverse transcribed with RevertAid MMuLV (Fermentas, Thermo Scientific) according to instructions.

Quantitative real-time RT-PCR analysis was performed on a Bio-Rad MyIQ Single-Color or CFX-Connect Real-Time PCR detection system (Bio-Rad, München, Germany) using Go Taq qPCR Master Mix (Promega, Mannheim, Germany). Primers were used at a concentration of 250 nM each. *Gapdh* was used as the reference gene. Following PCR program was used: 3 min at 95°C, 40 cycles of 15 seconds 95°C followed by 1 min 15 sec at an annealing temperature (58°C-63°C), 1 min 95°C, 1 min 55°C and melting curve cycle. Used primers had an efficiency level between 85% and 110%. Primer sequences are listed in Table S2.

qRTPCR results were analyzed using the ΔΔCt method [47]. Results were shown as fold change or Log_2_(fold change). The error bar was calculated based on the law of error propagation as previously described [45].

Viral RNA was isolated in RNAse-free conditions using a modified version of the protocol devised by Murdoch et al. [48]. Viral suspension was lysed using TRIzol reagent (Life Technologies). After adding 20 μg of glycogen (Peqlab, Erlangen, Germany) and bringing the suspension to a volume of 300 μl, 900 μl of TRIzol reagent followed by 240 μl of chloroform (Sigma) were added to the mix. Subsequent extraction and purification steps were carried out as originally stated [48]. 10 μl of the viral RNA were reverse transcribed, while other 10 μl were used for a mock-RT. RT and mock-RT products were diluted at least 1:10 and were used for qRTPCR using primers amplifying the Ψ packaging sequence. After setting up a standard curve, an absolute quantification was carried out.

The following formula was used to determine the concentration of lentiviral particles (expressed as LP/ml):

$$\text{Con}c_{\text{viral particles}}=\frac{\left(\text{copy numbe}r_{\text{vRNA}}-\text{copy numbe}r_{\text{plasmid}}\right)\times \text{Dilution factor}}{2\times V_{\text{start}}}$$

Where copy number*_vRNA_* is defined as the amount of vRNA determined from the reverse transcribed samples, *copy number_plasmid_* is the amount of plasmidic DNA carried over after transfection (determined from the mock-RT) and *V_start_* is the amount of viral suspension used for the quantification. We defined an IFU as 100 LP. The primers used are listed in Table S2.

### Gene expression profiling

Gene expression profiles of Foxg1^−/−^ mice (*n* = 3) and wild-type control mice (*n* = 2) were analyzed using Agilent's Whole Mouse Genome Microarray (026655). CPCs from E13.5 embryos were treated with ALK4,5,7-inhibitor SB431542 (10 μM) on DIV2 and on DIV3 treated with TGFB1 (5 ng/ml). CPCs were collected on DIV4 and total RNA was extracted and processed as described. See Figure S4 for a concise treatment scheme. Cy3 intensities were detected by one-color scanning using an “Agilent DNA Microarray Scanner (G250B)” at 5 μm resolution.

### Microarray data analysis

Intensity data were extracted using Agilent's Feature Extraction (FE) software (version 9.5.3.1) including a quality control based on internal controls using Agilent's protocol GE1107 Sep09. Microarray data analysis consists of the following steps: 1. between-array normalization, 2. global clustering and PCA-analysis, 3. fitting the data to a linear model, 4. detection of differential gene expression and 5. over-representation analysis of differentially expressed genes. To estimate the average group values for each gene and assess differential gene expression, a simple linear model was fitted to the data, and group-value averages and standard deviations for each gene were obtained. To find genes with significant expression changes between groups, empirical Bayes statistics were applied to the data by moderating the standard errors of the estimated values [49]. P-values were obtained from the moderated t-statistic and corrected for multiple testing with the Benjamini-Hochberg method [50]. For each gene, the null hypothesis, that there is no differential expression between degradation levels, was rejected when its false discovery rate was lower than 0.05. To find over-represented functions (as represented by Gene Ontology terms) [51], we used DAVID, the Database for Annotation, Visualization and Integrated Discovery [26, 27].

### Immunoblotting

The procedure was carried out as previously described [45]. Briefly, tissue or cultured CPCs were lysed in RIPA buffer (1% NP-40, 1% SDS, 0.5% sodium deoxycholate diluted in Phosphate Buffered Saline, PBS) supplemented with protease inhibitor (cOmplete Protease Inhibitor Cocktail, Roche) and sonicated for 10 cycles of 10 sec sonication and 10 sec pause with the Bioruptor Next Gen Sys (Diagenode, Seraing, Belgium). After centrifugation (10 min, 13000 rpm) the supernatant was collected. Protein concentrations were determined with Bradford reagent (Bio-Rad). 15 μg of protein extract were loaded with 5x Laemmli buffer on Mini Protean TGX gels (Bio-Rad) and run at 100V for 1.5 h. Proteins were transferred to PVDF membranes (Trans-blot Turbo Transfer Pack) using the Trans-blot Turbo Transfer System (both from Bio-Rad) following the manufacturer's instructions. Membranes were blocked with 5% BSA in TBS-T (blocking buffer) for 1 h and incubated overnight with primary antibodies (diluted in blocking buffer). Membranes were washed, incubated with secondary antibodies for 1 h and detected using ECL or Femto substrates (Thermo Scientific) and LAS ImageQuant System (GE Healthcare, Little Chalfont, UK).

The following antibodies were used: anti-FOXG1 (1:1000 dilution; #18529) and GAPDH (1:3000 dilution; #8245), from Abcam. For densitometric analyses, ImageJ software was used [52].

### Immunohistochemistry (IHC) and immunocytochemistry (ICC)

IHC was performed on fixed forebrains (4% PFA) embedded in Tissue Freezing Medium (Leica biosystems, Wetzlar, Germany) and cut into 12 μm section. Prior to their use, sections were thawed for 10 min at 37°C and subsequently postfixed for 30 min. Permeabilization was performed in either 0.1% or 1% Triton X/PBS for 30 min. After 1 h of blocking in 5% BSA/PBS (Roth, Karlsruhe, Germany) or in 1% BSA/4% donkey serum/1% normal goat serum (NGS)/0.1% Triton X/PBS, sections were incubated with primary antibody against CALB2 (#92341, 1:100 in 1% BSA/PBS, Abcam) or FOXG1 (#18529, 1:500 in 1% BSA/4% donkey serum/1% NGS/0.1% Triton X/PBS, Abcam), respectively. On the next day, secondary antibody was applied for 1 h at RT. Sections were then counterstained with 1:1000 4′,6-diamidino-2-phenylindole (DAPI) solution and embedded in Fluorescent Mounting Medium (DAKO, Jena, Germany).

For ICC, cells plated on coverslips were fixed for 20 min with 4% PFA and permeabilized in acetone for 10 min at −20°C. Blocking was performed in 10% normal goat serum, 1% Triton X/PBS for 1 h at RT, followed by the incubation with primary antibodies in blocking solution at 4°C overnight. The incubation with secondary antibodies was carried out for 1 h at RT, followed by counterstaining with DAPI (1:1000) and mounting in Fluorescent Mounting Medium (DAKO). The following antibodies were used: Anti-HuC/D antibody #A-21271, 1:100 (Life Technologies), FOXO3 #47409, 1:500 (Abcam). All secondary antibodies were purchased from Invitrogen.

Images were taken with Axioplan 2, Axio Imager M2 with or without ApoTome 2.0 (Zeiss, Jena, Germany). Positive cells were counted manually using ImageJ software [52]. For each condition, at least 947 total cells were counted.

### Chromatin immunoprecipitation

The procedure was carried out as previously described [53] with some modifications. CPCs were fixed with 1% freshly prepared PFA (Roth) for 5 min at RT. The cross-link was stopped by addition of glycine to a final concentration of 125 mM. Cells were washed twice with PBS and lysed in DNA lysis buffer (50 mM TRIS-HCl, pH 8.0, 10 mM EDTA, 0.1% SDS) supplemented with protease inhibitor (Roche). Chromatin was sheared within 30 high-energy cycles by the Bioruptor Next Gen System (Diagenode): 30 s on and 30 s off to achieve DNA fragment lengths between 200-600 bp. The chromatin concentration was quantified with the NanoDrop 2000 (Thermo Scientfic), diluted in DNA dilution buffer (20 mM TRIS-HCl, pH 8.0, 150 mM NaCl, 2 mM EDTA, 1% Triton X-100) supplemented with protease inhibitor (Roche), bovine serum albumin in PBS (final concentration: 0.1%) and precleared using Protein A Dynabeads (Life Technologies) for 2 h at 4°C. Antibodies were bound to equilibrated Protein A Dynabeads for 2 h at 4°C. Precleared supernatants were then added to antibody-bound beads. For immunoprecipitation, 20-30 μg of chromatin were incubated either with antibody directed against FOXG1 (1 μg per 20 μg chromatin; #18529, Abcam) or rabbit IgG (1.3 μg per 20 μg chromatin; #C15410206, Diagenode) at 4°C overnight. Immunocomplexes were washed for 10 min once with buffer 1 (20 mM TRIS-HCl, pH 8.0, 150 mM NaCl, 2 mM EDTA, 1% Triton X-100, 0.2% SDS), buffer 2 (20 mM TRIS-HCl, pH 8.0, 500 mM NaCl, 2 mM EDTA, 1% Triton X-100, 0.2% SDS), buffer 3 (20 mM TRIS-HCl, pH 8.0, 250 mM LiCl, 2 mM EDTA, 1% NP-40, 1% sodium deoxycholate) and thrice with TE buffer (20 mM TRIS-HCl, pH 8.0, 2 mM EDTA) at 4°C. DNA was extracted from beads in extraction buffer (1% SDS / 100 mM NaHCO_3_) by shaking at 1400 rpm at RT for 1 h. ChIP and input DNA samples were incubated with RNase A (Sigma) at 37°C for 30 min, followed by the Proteinase K (Roche) digestion at 65°C while shaking at 1400 rpm for 6 h during the removal of the cross-link. Purification of the ChIP and input DNA samples was done following the instructions provided by the manufacturer of the MinElute Reaction Cleanup Kit and PCR purification Kit (both Qiagen), respectively. All DNA samples were diluted accordingly, quantified fluorospectroscopically using the Quanti-iT-PicoGreen dsDNA Assay Reagent (Life Technologies) and the NanoDrop 3300 (Thermo-Scientific). To analyze the enrichment of the ChIP samples, a qPCR was performed. 20 to 320 pg of DNA was applied per reaction. Ct values of ChIP samples were normalized to Ct values of input to calculate enrichment (% input). Final results are shown as a ratio between FOXG1 enrichment at a specific site and its enrichment in a region devoid of FOX binding sites, which was used as a negative control.

### Riboprobe synthesis and *in-situ* hybridization

This experiment was performed as previously described [54] with some modifications. Briefly, PCR products using *Kcnh3* primers (forward: CAGCTTTATGGACCTCCACTTC; reverse: AGAGCCTGTGGATCTCTAGCC, Allen Brain Atlas [55, 56]) were cloned into the pGEM-T Easy Vector System (Promega) according to manufacturer's instructions. The cloning product was linearized through digestion with NcoI (sense probe) or SacI (antisense probe) and transcribed with SP6- or T7-RNA polymerases (Roche), respectively. Forebrain slices were hybridized with digoxigenin-labelled riboprobes in hybridization buffer (12.7 mM Tris base, 184.4 mM NaCl, 5.9 mM NaH_2_PO_4_, 6.27 mM Na_2_HPO_4_, 5 mM EDTA pH 8.0, 0.5x Denhardt's solution, 1 mg/ml Yeast RNA, 10% Dextran sulfate, 50% v/v Formamide) at 68°C overnight. Sections were washed thrice in a solution containing 50% formamide, 0.1% Tween-20 and 5% saline sodium citrate at 68°C in a water bath. They were then transferred to an incubation chamber and washed twice with maleic acid buffer and Tween-20 (MABT) for 30 min at RT. After blocking in a MABT solution containing 20% lamb serum, sections were incubated with an alkaline phosphatase-conjugated anti-digoxigenin antibody (1:1500 in blocking solution; Roche) overnight at RT. After four washing steps in MABT (10 min, 3x 20 min) and three washing steps (7 min each) in pre-staining buffer, the reaction product was developed using NBT/BCIP solution diluted in pre-staining buffer (1:100; Roche) overnight at RT. Stained sections were washed 4 times in PBS and then embedded using Aquatex (Merck Millipore). Image acquisition was performed using an Axio Imager 2 microscope without apotome (Zeiss).

### Statistical analysis

Unpaired Student's *t*-test was used for all qRTPCR experiments where mutant values were compared with their respective controls and, in general, when comparison was performed between two groups. One-way ANOVA with either Šidák's, Dunnet's or Tukey's post-test was used in experiments involving comparison among more than one group. Analysis of experiments involving cell infection with lentiviral shRNA constructs and ChIP was performed using One-sample *t*-test, where comparison was made between each value and the control value (set as 0). Values in bar charts are expressed as average ± SEM.

## SUPPLEMENTARY MATERIAL FIGURES AND TABLES




