# Supplementary material for: The FOXG1/FOXO/SMAD network balances proliferation and differentiation of cortical progenitors and activates Kcnh3 expression in mature neurons
Source: Oncotarget. 2016 May 21;7(25):37436–55. doi: 10.18632/oncotarget.9545 (PMC5122323; doi:10.18632/oncotarget.9545)
Supplement: Supplementary file 1 [file oncotarget-07-37436-s001.pdf]

The FOXG1/FOXO/SMAD network balances proliferation and differentiation of cortical progenitors and activates *Kcnh3* expression in mature neurons

Supplementary Material

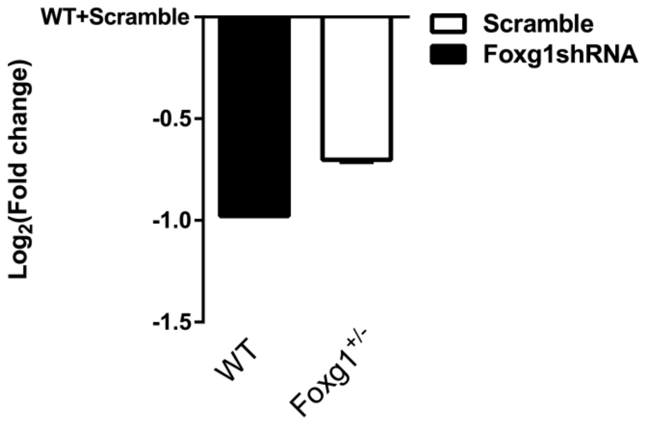

Fig. S1: Knockdown efficiency: comparison between shRNA -mediated knockdown and *Foxg1* heterozygous mice. Transcriptional levels of *Foxg1* were compared (using qRTPCR) in E13.5 wild-type CPCs infected with a *Foxg1*-specific shRNA construct and *Foxg1*-heterozygote animals infected with unspecific scrambled shRNA control. The observed decrease in *Foxg1* expression was similar in both conditions as compared to control condition (wild-type E13.5 CPCs infected with scrambled shRNA construct). n=1 for wild-type condition; n=3 for Foxg1$^{+/-}$ condition.

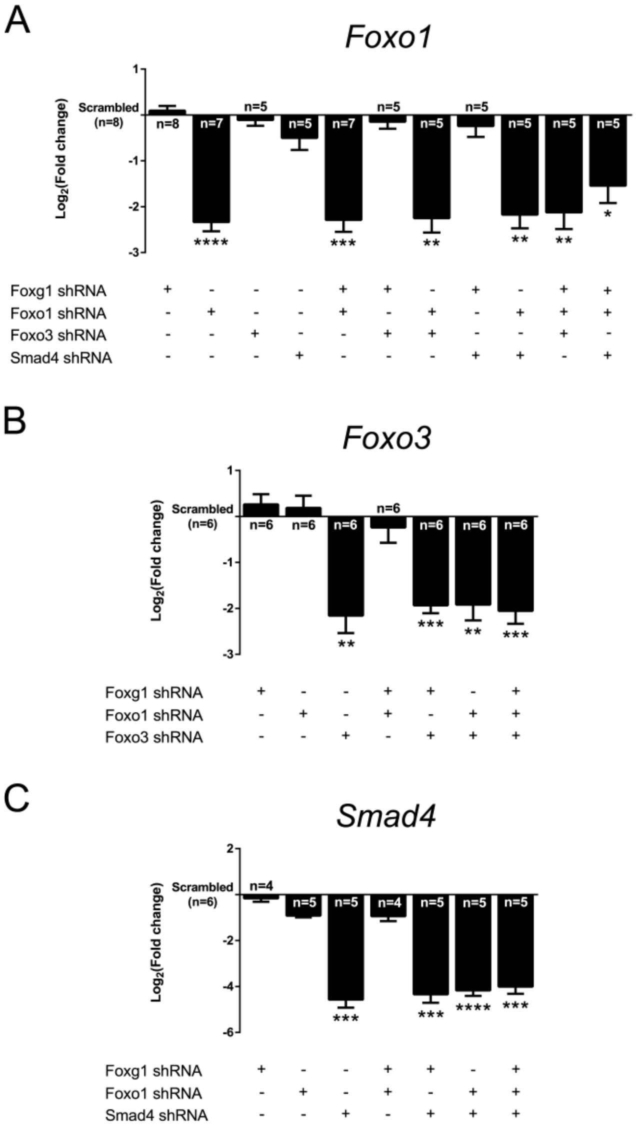

Fig. S2: Validation of shRNA-mediated knockdown for *Foxo1, Foxo3* and *Smad4.* A-C E13.5 murine CPCs were infected with shRNA constructs targeting specific genes or scrambled shRNA construct (control). Expression levels *of Foxo1, Foxo3* and *Smad4* were assessed by *qRTPCR.* **A** *Foxo1* transcript levels were reduced only when shRNA targeting *Foxo1* was employed. **B** *Foxo3* expression was only significantly inhibited when *Foxo3* shRNA constructs were used. **C** *Smad4* expression was significantly repressed upon *Smad4* knockdown. Results are shown as mean of Log_2(fold change)±SEM in specific shRNA construct condition vs. scrambled control (set as 0). ****$p<0.0001$, ***$p<0.001$, **$p<0.01$, *$p<0.05$; One-sample t-test; replicate numbers indicated in graphics.

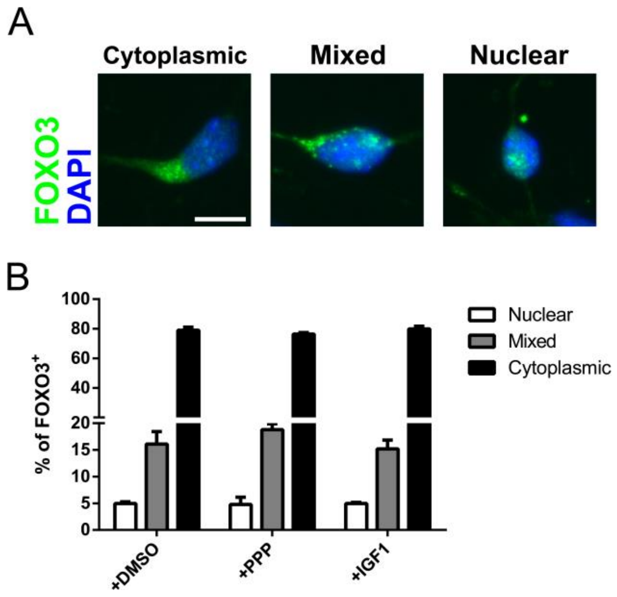

Fig. S3: Assessment of nuclear localization of FOXO3 after treatment with IGF1-pathway inhibitor or IGF1. A E11.5 CPCs were cultured for 4 days, treated with either the IGF1-signaling inhibitor picropodophyllin (PPP) or with IGF1 for 30 min prior to fixation. Immunocytochemical staining revealed that neither PPP- nor IGF1-treatment affected the intracellular localization of FOXO3 in a significant manner. Statistical analysis: One-way ANOVA; Šidák's post-test. n=3. Scale bar: 10 µm.

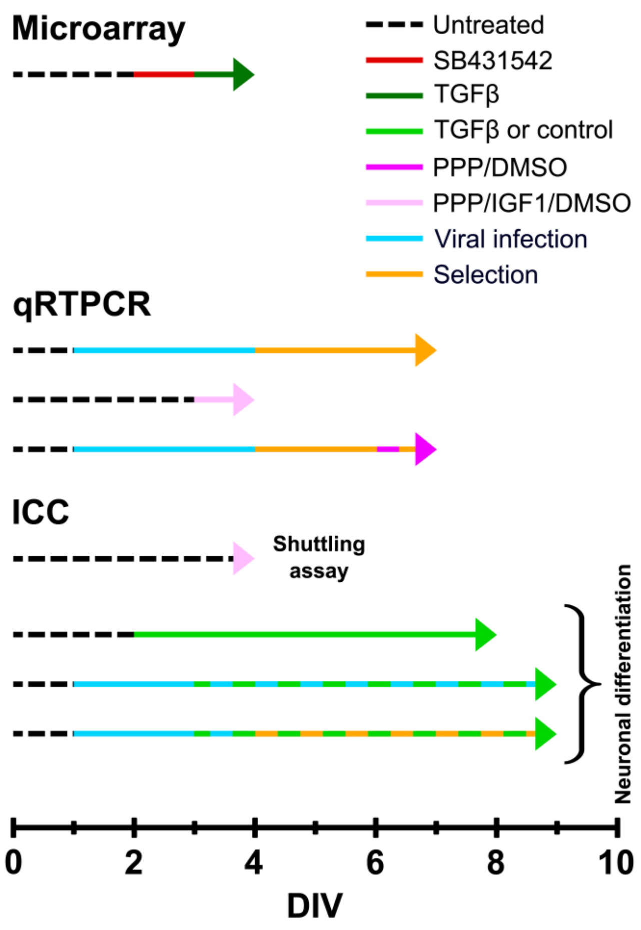

Fig. S4: Treatment scheme for various CPC culture experiments.

Supplementary Table S1: List of genes differentially expressed in TGFβ-treated *Foxg1*$^{-/-}$ as compared to TGFβ-treated wild-type CPCs. The table is provided in an additional file.

Supplementary Table S2: List of primers used in qRTPCR and ChIP.

Primer name	Forward (5'-3')	Reverse (5'-3')	Use	Ref.
A930038C07Rik	GCTTTTTCCGCACCACACA	GTGAGTGGTAACAGGAGGCA	qRTPCR	
Bst2	CGAGACACAGGCAAACTCCT	TCCTGGTTCAGCTTCGTGAC	qRTPCR	
Calb2	GATGAGAATGAACTGGACGCC	GGCCAAGGACATGACACTCT	qRTPCR	
Cdkn1a	CGGTGTCAGAGTCTAGGGGA	AGAGACAACGGCACACTTTG	qRTPCR	
Cdkn1a -2287bp	GTCACAGCACTCAGCAGTTC	AAACTGCCCAAATCCCCTTG	ChIP	
Chchd2	TCCACCCTTCCTCTCTCCT	TGAGCTGCAGGTGCTCTTC	qRTPCR	
Cln5	ACGACTACAAGCCCCGATTT	TCAGTGTGCTCCTGAATCCC	qRTPCR	
Cryab	ACCTCTTCTCAACAGCCACTT	TGCTTCACGTCCAGATTCAC	qRTPCR	
Ecm2	ACAGGACTATCAGCTGCGTT	ACCTATGCCCGAAGAAGTGA	qRTPCR	
Fgd6	GAACAACATGCTCTCGCTGG	GCCAGTCATCTCTTTCTGCG	qRTPCR	
Foxg1	AATGACTTCGCAGACCAGCA	CCGGACAGTCCTGTCGTAAA	qRTPCR	
Foxo1	ACTTCAAGGATAAGGGCGACA	CCTCCCTCTGGATTGAGCATC	qRTPCR	
Foxo3	GCCTCATCTCAAAGCTGGGTA	CGGATCACTGTCCACTTGCT	qRTPCR	
Foxo4	CCCTACTTCAAGGACAAGGGT	GCCTCGTTGTGAACCTTGATG	qRTPCR	
Igf1	GTGGATGCTCTTCAGTTCGTG	ACTCATCCACAATGCCTGTCT	qRTPCR	
Igf1r	ATGACACGCGGTGATCTCAA	TGCCATCTGCAATCTCTCCA	qRTPCR	
Igf2	GCTTCAGTTTGTCTGTTCGGA	GGGGTGGCACAGTATGTCT	qRTPCR	
Igf2bp1	CGTCACCTACTCTAACCGGG	AGGACCTTGCGTTATCTGCT	qRTPCR	
Igf2bp2	GACCCTCTCGGGTAAAGTGG	CCATATTCAGCCAACAGCCC	qRTPCR	
Igf2r	AAGGCAGTCTCATCATGCCA	ACTGGCTTCTGTGTAGTCCG	qRTPCR	
Igfbp2	CTACGCTGCTATCCCAACCC	CTGCTACCACCTCCCAACAT	qRTPCR	
Igfbp3	GAGTCTAAGCGGGAGACAGAA	CCCATACTTGTCCACACACCA	qRTPCR	
Igfbp4	ATTCCAAACTGTGACCGCAAC	CCCTGTCTTCCGATCCACAC	qRTPCR	
Igfbp7	CAAGGTGTTCTTGAGCTGTGA	AGCCCGTTACTTCATGCTTTTC	qRTPCR	
Kcnh3 -1428bp	GGATTTCAGGGTGCAGCAGA	ACCCGTCAGCAGCTAGTAAA	ChIP	
Kcnh3 3'-UTR	CCATTCCTTGCATGTGCCC	TCCAGTGAGTCAGCTTTAGACT	ChIP	
Kcnh3	AACACCTTCCTGGACACCAT	GTAGACCACAGGGAAGAGCC	qRTPCR	
Lenti ψ	AGCGAAAGGGAAACCAGAGG	GCACCCATCTCTCTCCTTCT	qRTPCR	
Ltbp1	CTGGGGAGACAACTGTGAGA	CCACCAGTGTCGTAAGAGGA	qRTPCR	
Negative Ctrl.	GGGGGATAATGATTGCAAAA	GCGTGGACAGAGATCTAGGC	ChIP	[1]
Nell2	CTGCGTATGTGGATGGCAAG	GGACAATCTAAGGGTGGGCA	qRTPCR	
Parp4	CCACGACGATGAAACCAACC	CTGGTTGCTTTTCCTGCCAA	qRTPCR	
Pcdh7	CTGGGCGTCTCTGAAGATGG	TGAAAAATTCCACGGCAGGTC	qRTPCR	
Plxdc2	GATTTGATCGCCATCGGCAG	AATTGCATGGTGGTCGTGTG	qRTPCR	
Pou3f3	GAAGGGTAGGTACGTCCAGC	GGCGGAATCTTTAGGCATGG	qRTPCR	
Reln	TGGCAACCCATCCTTCCACCTCT	CACGAGCTGCCAGGAATCGGAC	qRTPCR	
Rnd2	GTTATGTCCCCACGGTGTTT	ACAGCATCAGAATCCGGGTA	qRTPCR	
Rspo2	CATTGCGGAGTCCAGGAGAT	CACTCTGTCTGTAGCGAGGA	qRTPCR	
Smad2	CCCTTCAGTGCGATGCTCA	GAATACTACGACGGAGGAGCTGTT	qRTPCR	
Smad3	CACGCAGAACGTGAACACC	GGCAGTAGATAACGTGAGGGA	qRTPCR	
Smad4	ACAGAGAACATTGGATGGACGA	ACGGGCATAGATCACATGAGG	qRTPCR	
Smad7	GGCCGGATCTCAGGCATTC	TTGGGTATCTGGAGTAAGGAGG	qRTPCR	

Tgfb1	GCCTGAGTGGCTGTCTTTTG	GGCTGATCCCGTTGATTTCC	qRTPCR	
Tgfb2	CCCTTGGAAAACAAAACAGGTG	CCCCCTGGCTTATTTGAGTTC	qRTPCR	
Tgfb3	GCAAGAATCTGCCCACAAGG	CCATTGGGCTGAAAGGTGTG	qRTPCR	
Tgfbr1	AAGTTTGGCGAGGCAAATGGCG	TCTGACACCAACCACAGCTGCG	qRTPCR	
Tgfbr2	AGAAAGATGCATCCATCCACCT	CATGCAGGACTTCTGGTTGTC	qRTPCR	
Tgfbr3	CCTGACGGGGGCCTTGTGGT	TATGCTGTGGGCTGCGCTGC	qRTPCR	
Trp73	CATCTGTTGAGCAAGGGCAG	TAGAGCTGTCAGGCACGGTA	qRTPCR	

SUPPLEMENTARY REFERENCES

1. Webb AE, Pollina EA, Vierbuchen T, Urbán N, Ucar D, Leeman DS, et al. FOXO3 shares common targets with ASCL1 genome-wide and inhibits ASCL1-dependent neurogenesis. Cell Rep. 2013;4:477–91.
